# Editorial: The roles of genome and epigenome in poorly studied male-specific cancers

**DOI:** 10.3389/fonc.2023.1166320

**Published:** 2023-03-22

**Authors:** Rong Na, Qiyuan Li

**Affiliations:** ^1^ Department of Surgery, Li Ka Shing (LKS) Faculty of Medicine, The University of Hong Kong, Hong Kong, Hong Kong SAR, China; ^2^ National Institute for Data Science in Health and Medicine, School of Medicine, Xiamen University, Xiamen, China

**Keywords:** cancer, male, prostate cancer, Paget disease, breast cancer

Male-specific cancers, such as prostate cancer, male breast cancer, testicular cancer, etc., have become one of the major health issues worldwide (1). Except for prostate cancer, most of the other male-specific cancers are less studied compared to their female-specific counterparts (i.e., breast cancer, ovarian cancer). More and more recent reports suggested that male-specific cancers carry characteristic changes in the genome due to sex differences(2–4). We, therefore, initiated this special topic to discuss these under-reported diseases. The topic contains 4 original research articles and 1 review article which have involved different male-specific malignancies, including prostate cancer, male Paget disease, and male breast cancer.

Johnson et al. presented a comprehensive review regarding the genetic contributions to prostate cancer in Men with West African ancestry. As one of the least developed regions in the world, very little evidence could be obtained regarding the disease characteristics. In this review, the authors discussed the potential disparities between West African ancestry and other ancestries (in particular, the general African American population). In addition to the evidence provided by this review, it may also bring more attention from the readers to the healthcare issues in under-represented populations in the world.

Two research articles provided new classifications of prostate cancer using different genetic signatures (Cheng et al., 2021;Yu et al., 2022). One of the studies presented a subgroup of prostate cancer with significant enrichment of genetic variants in metabolism-related genes (Yu et al., 2022). Another study identified a subgroup of prostate cancer with a poorer prognosis which embraces significant expression variations in immune-related genes (Cheng et al., 2021). The results provided different angles in understanding the biological mechanisms of prostate cancer.

Penoscrotal extramammary Paget disease is a rare and poorly studied malignancy in men. Though most male Paget diseases are indolent (but commonly recurrent), some cases could have very invasive phenotypes. Rao et al. performed whole exome sequencing in six paired invasive vs. indolent (*in situ*) penoscrotal extramammary Paget diseases in Chinese male patients and found several potential genes that might contribute to the disease progression. This research article provides important new evidence to prompt further in-depth research into this poorly understood disease.

In sharp contrast to female breast cancer, for which thousands of research articles have been published, male breast cancer has been less studied. Last but not least, a group of physicians and scientists from Italy reported their most recent results on this topic(Valentini et al., 2022). As one of the two studies explored the genomic landscape of male breast cancer to date, this research article would no doubt provide important information for us to better understand the mechanism of carcinogenesis, as well as to provide better disease management with precision medicine approaches. The results from this study also revealed the potential differences between male breast cancer and well-studied female breast cancer, which might raise the research interest in this topic among the readers for further investigation.

In summary, these five articles collected on the current Research Topic not only provide scientific values, but also (and this is more important) draw researchers’ attention to these under-represented diseases, known as male-specific cancers.

## Author contributions

All authors listed have made a substantial, direct, and intellectual contribution to the work and approved it for publication.
